# Surgery

**Published:** 1860-03

**Authors:** 


### SURGERY.

1. On the Great Importance of Early Diagnosis and Treatment for Stone in the Bladder. By *Henry Thompson, F.R.C.S.*, Assistant Surgeon to University College Hospital, etc. etc.—There are few among the maladies which afflict humanity that are generally regarded, both by the patient and the surgeon, as fraught with graver import than stone in the bladder. There are no cases that are observed in our hospitals with greater interest, and there are none in which the surgical procedures commonly adopted for their relief involve greater responsibility on the part of the operator, or are more attractive to the surgical student, from the momentous importance which attaches to them. Grave in the highest degree, however, as by universal consent is the situation of the adult patient with a calculus of considerable size in his bladder, it is a fact worthy to be well and deeply considered, that the existence of such a calculus is, in the great majority of cases, a matter wholly preventible; and that, within certain limits, it must be regarded as a result which, by the exercise of a moderate amount of intelligence and care, may be rendered extremely uncommon. It is so, because the presence of a vesical calculus may almost always be ascertained in an early stage; and because its destruction in that stage is an operation which, with a fair amount of skill, may generally be accomplished with certainty, and with safety to the patient. The first indication—namely, to prevent the formation of a stone of moderate or average size—is insured by an intelligent appreciation of the early signs which it produces; in other words, by a diagnosis of its existence while it is yet quite small. The second indication may generally be fulfilled by crushing it in that stage.

In reference to the subject of counteracting a constitutional tendency to calculous formation, it is not within the limit of this article to enter on the important and fertile theme of medical treatment for its prevention. The question here is only that of early diagnosis, and the treatment which is desirable when the existence of a small stone is verified. Before entering on this, I shall cite briefly a few cases in illustration of the proposition, that under these circumstances the cure of the complaint is safe and simple.

*Case* 1.—A gentleman, aged thirty-nine, while staying in Malta on his return from India, was recommended to consult me by my friend Mr. Filliter, then in practice there. He had suffered for some months from irritability of the bladder and severe pain after passing urine, with occasional pains in the back and loins. There had been no bleeding observed, and no pain in the glans penis. Instruments had been passed in India; no stricture was discovered. The general health was somewhat impaired. I saw him first on October 16th, 1857, and after investigating his case suspected the presence of stone. It was agreed that he should be sounded in a few days. The urine exhibited on two occasions the presence of blood, although in quantities only to be appreciated by the microscope.

Oct. 24th.—The pelvis being well elevated, I introduced a lithotrite; and, after some little searching, caught a small stone, and effectually crushed it. He was in bed, and remained so during the day.

25th and 26th.—Irritability increased; several fragments came away, which he carefully preserved. From their appearance I was inclined to think there must have been two small calculi.

29th.—Much better. I introduced the lithotrite and found one fragment, which was crushed.

Nov. 1st.—More debris has been passed.

15th.—Perfectly free from all symptoms.

*Case* 2.—A gentleman, aged thirty-one years, was brought to consult me by my friend Mr. Tweed, of Brook Street, on September 8th, 1857. During the last eighteen months he has suffered severely from urinary derangement; and for fifteen months there have been fistulæ in the perinæum. In France, where he resided during the summer, he was advised to pass all his urine by catheter, in order to give them a chance of closing. He did so, and with the desired effect. They have since, however, reopened, and they exist now. On examination, no stricture nor any disease of the prostate is detected, and there appears no reason to believe that either has existed. I then introduced a sound, and discovered a small calculus.

The next day, September 9th, he paid me a second visit, when I introduced a lithotrite, and crushed at once a small calculus about the size of a bean. He walked home to his house in Brook Street.. During the week he passed debris, which were all phosphatic.

Sept. 16th.—I introduced a lithotrite, but could find nothing in the bladder. Symptoms continue, although diminished in intensity.

22d.—Again introduced the lithotrite, found a small piece and crushed it. The debris passed next day.

Oct. 1st.—He left town perfectly free from symptoms; but he passed a few small fragments during the month.

After this he completely recovered, and enjoyed excellent health for more than a year. He had, however, wholly neglected to adopt a system of diet and medicine which we were anxious he should follow perseveringly, as there was reason to believe a constitutional tendency to form phosphatic matter existed, the urine being still always alkaline and phosphatic.

In May, 1859, he reappeared with a calculus of considerable size, and in completely broken health, having suffered severely upwards of a year. After due preparation, I attacked the calculus, and successfully removed it after six applications of the lithotrite. He is now enjoying excellent health. The fact only is mentioned here, as the former part of the case illustrates the subject of this paper.

*Case* 3.—A gentleman, aged forty-five, was under my care in September, 1858, for stricture of the urethra, which was successfully treated by dilatation in the ordinary manner. Some symptoms of urinary derangement remained after No. 11 sound had been reached, which evidently demanded further investigation. Accordingly, I introduced a lithotrite, and, after some searching, discovered a calculus measuring three-eighths of an inch, according to the scale of the instrument. I crushed it at once, at the patient's rooms.

Next day, October 12th, some fragments of urate of soda passed, and debris followed on succeeding days.

Oct. 19th.—Again crushed two or three fragments. After three days no more debris passed, and the symptoms disappeared. A week afterwards the patient was carefully sounded, but nothing was discovered. He has been perfectly free ever since. The calculus was urate of soda.

*Case* 4.—H. A., aged thirty-six, and supposed to be the subject of stricture, was sent to me as an out-patient at University College Hospital, February 25th, 1859, with some symptoms, not very urgent, of urinary derangement. I passed an instrument, and found that there was no contraction or other cause for his complaints in the urethra or prostate, and then introduced a sound into the bladder, and soon discovered a small stone. His urine was examined, found healthy, but depositing octahedra of oxalate of lime. He was directed to come at my next visit, March 1st, when I introduced a lithotrite, and in the presence of a number of pupils, without any pain worth mentioning, as he said, and certainly without any expression of it, I crushed at once a calculus about the size of a nut. It is almost unnecessary to say that he had no chloroform. There were no traces of bleeding, and no signs of irritation following. Consequently, after an hour's rest, I desired him to walk slowly, not to ride, to Camden-town, where he lived. Two succeeding crushings followed—one on the 6th, the other on the 9th of March, small debris passing easily after each sitting. The calculus was oxalate of lime. Being perfectly relieved of all his symptoms, he returned to his work at Pickford's, as night-porter, on the 16th of March, being the fifteenth day from his first sitting. He has continued at work ever since. and is perfectly free from all his old symptoms, having called at the hospital during the last month to tell me so

It is by no means uncommon to find small calculi forming in connection with stricture of the urethra. These may be spontaneously voided after dilatation has been fully completed, or their removal may be accomplished by some mechanical aid from the surgeon. Such occurrences are to be looked for when symptoms persist after the urethral obstruction has been removed. By so doing we may be the means of freeing the patient from the presence of a small concretion, and may probably save him from the grave consequences of serious calculus formation hereafter. I have several such small calculi, which have been removed or expelled from different patients in such circumstances. Of these, the following case is an interesting example:—

A gentleman was recently brought to me with an extremely obstinate stricture, by Mr. Davis, of Hanley, Staffordshire, who stayed with him during the treatment. This consisted, at first, in introducing a very slender silver catheter, which, after some time and trouble, I succeeded in accomplishing; in tying it in, and subsequently replacing it by gum catheters, also tied in, until a tolerable size had been reached. During the progress, a symptom or two occurred which led us to suspect a small calculus, and on one occasion I was satisfied that I had felt it with the gum catheter. Next day I engaged it in the eye of the catheter, and drew it along the urethra as far as to the stricture, beyond which it could not be made to pass. Twenty-four hours more of tying-in dilated this still further, and next day it was again caught, and ultimately came away. It was about the size of a grain of wheat, but longer. Had it not been removed in this early stage, it would undoubtedly have formed the nucleus of a larger calculus, and it is on this account mentioned here as another illustration of the principle laid down.

It appears to me to be desirable also, in the cases of children, to ascertain if possible, at an early period, the existence of calculus. A very small one, having made its descent from the kidney to the bladder, instantly gives rise to symptoms in young subjects; and before it reaches the size of an apple-pip, is often the cause of great distress. Many, doubtless, have seen the operation of lateral lithotomy performed for a concretion even smaller than that. In such a case I prefer a small lithotrite, of which the diameter is between five and six of the ordinary catheter scale, made for me by Mr. Coxeter; it does not require a screw, which simplifies the instrument greatly, the power applied by simple pressure of the hand being amply sufficient for such purposes. With this the stone is seized, and if the scale indicates that its size is very small, it may be crushed at once; if it is larger than a pea, I think it is better to cut, as the expulsion of any but small fragments is attended with much difficulty, while the cutting operation is generally as happy in its results in children as it is hazardous in the adult. After lithotrity in them, the efforts of the bladder are very powerful, while the urethra is so narrow and delicate that, except in the smallest calculi, the removal of fragments may occasion much difficulty, and then, no doubt, lithotomy, especially the median, would be both simpler and safer. The great object, however, should be to detect the calculus when it is, as all are at first, extremely small.

The application of the principle is easy; a case which occurred about a month ago, at University College Hospital, will illustrate this:—

A little boy, aged three years and a half, was brought to me at the out-patient's room on the 30th of November, 1859, with symptoms of urinary derangement, said to be of four months' duration. He had been under medical treatment two months without benefit. Suspecting calculus, he was put under chloroform. I sounded him, and found one which, from the bladder having expelled its contents, appeared to be small. He was desired to come again in a week's time, intending, if it proved to be so, to crush it.

Dec. 9th.—Under chloroform, I introduced the small lithotrite and caught a stone which measured half an inch in diameter. I decided at once to make no attempts to crush it, but to cut him, which I did on the 14th by the median operation, removing a stone the size of a marble. He made a rapid recovery, and left the hospital on the 30th.

Had the stone been half the size, I could easily have reduced it to powder with the small lithotrite—the essential proceeding in the case of children—its flat blades, not fenestrated, being especially adapted for reducing fragments to a state of powder, which passes easily.

It is important to inquire what are the signs which indicate the existence of calculus in that form of small concretion which it must always assume in the early stage, and which in most cases it maintains for a considerable period of time during the early history of its progress. The ordinary and most obvious symptoms of stone are usually regarded as the following: Pain at the close of the act of passing urine; pain in the glans penis; pain increased by sudden movements of the body; sudden stopping of the stream of urine during its flow, and its reappearance after change of position in the body; occasionally blood in the urine after exercise. In addition to these are frequent wants to micturate—a symptom met with more or less in all complaints of the urinary organs. In most cases, also, there is some change in the urine itself, produced by organic or inorganic additions to the normal elements. In elderly subjects with enlarged prostate, the urine sometimes affords more marked evidence than the subjective symptoms, which may be masked by the condition of the prostate, or confounded with the symptoms arising therefrom. Among the numerous patients who complain chiefly of irritability of bladder, and some pains associated with micturition, we may rely upon it that in nineteen out of twenty there is some material and recognizable cause. There is either inflammation of some portion of the track from the kidney to the urethra inclusive, the locality of which may be determined; or there is obstruction, the precise nature of which can be ascertained; or there is some adventitious growth or deposit, or some organic change taking place in some part, and presenting symptoms or imparting characters to the urine which are almost unmistakable. Now, in the early stage of vesical calculus, its discovery is due at first to the observation of the negative rather than of the positive signs. Frequent and painful micturition being complained of, the absence of inflammation of the urethra or bladder, or, at all events, its non-existence as a primary state, is ascertained by reference to the present condition and to the history; while catheterism determines the absence of obstruction; and urinary examination, in connection with the general symptoms, indicates whether or no organic disease exists. Having thus excluded all these causes, the occurrence of marked pain at the end of micturition, the occasional appearance of a little blood in the urine, although only microscopic, with the persistence of some crystalline deposit and of some pus, however slight in amount, in the urine, should awaken a strong suspicion of the existence of a calculus. A careful examination, by the microscope, of the urine passed on several successive days should generally be made, always in cases of obscurity, since the indications just alluded to, and which are so obtained, are often extremely valuable. If, in addition to these signs, there is any distinct history of recent pain, more or less acute, in the course of either ureter, and lasting for a few hours, the suspicion will be greatly strengthened. Whether this last-named sign have been present or not, a patient in the circumstances above described ought to be sounded. In the operation of searching for the calculus itself, it is necessary to remember that a small stone is less easy to detect than one of ordinary size, and that it is as difficult sometimes to miss the one as to find the other. In order to sound efficiently, but especially in a case where there is reason to believe that the stone is small, a sound of appropriate form is necessary. It is barely possible to search a bladder thoroughly with a sound curved like the ordinary catheter, inasmuch as every part of the cavity cannot be explored by such an instrument. The sound should have a very short and well-curved extremity, which can be rotated in the bladder without difficulty, and without causing pain to the patient, so that the floor of the bladder behind the prostate can be searched as freely as any other part by turning the short beak downward. It is manifestly impossible thus to explore if the curved portion of the sound be long, and the defects of such an instrument are still more obvious when the prostate is materially enlarged. Next, the sound should be hollow, so that water can be injected through it into the bladder, or flow outward from it, in order that the surgeon may possess the advantage of sounding while the bladder is in various degrees of distention; indeed, in every intermediate condition between full and empty. The patient should lie on his back, with the pelvis elevated from four to six inches above the shoulders. If nothing is found, supposing the search to have commenced with about ten ounces of fluid in the bladder, about half may be allowed to run off as the searching continues. At this point, if nothing is discovered, the patient may be carefully raised into the upright position, and the bladder then be permitted to empty itself through the sound, which, being gently moved, will scarcely fail to detect a very small calculus, if such a one be present, since it is almost certain to be brought down against the sound with the last portion of the outflowing current.

Perhaps the best situation for the eye of a hollow sound is on the convex part of the curve, and not at its extremity, which, being thus left solid and heavy, affords more certain indication to the operator than if hollowed, and consequently feeling light in the hand. The material advantage gained by employing the hollow sound is, that the introduction of a single instrument only, suffices for the whole process of emptying, injecting, and searching the bladder. The lithotrite itself answers very well as a sound; and, if it possesses a small channel through it so as to admit the flow of fluid, is equal to the instrument just described, with the single drawback of its being less rounded in its surface and outline, and thus a little less easily borne by the patient. On the other hand, it enables the operator to crush a small calculus at the same time, without further manipulation, if thought desirable.

It is my firm belief that the early diagnosis of calculus might, in the great majority of cases, be made without difficulty, if suspicious symptoms were more sedulously observed when they first arise, and the necessary examination made when simple means had failed to dispel them. For it may be safely affirmed that there are very few patients indeed, who will not seek professional advice against the troubles which arise during the early stages of the formation, these being quite sufficient to awaken their anxious attention. Granting the truth of this proposition, and holding the certainty and safety of lithotrity for small calculi as a universally admitted fact, there inevitably results the following deduction, the importance of which it is impossible to over-estimate, namely:—

That stone in the bladder—one of the direst of human maladies, and in the removal of which one of the most severe and fatal operations is still most commonly performed—may, through the exercise of ordinary intelligence and skill, be deprived of nearly all its severity and of its danger to life.

I cannot doubt that the time is not very far distant when the gravity of the affection will in this manner be greatly diminished. It will be so in proportion to the increased vigilance which is exercised in practice. Much as our art accomplishes in the cure of established disease, its value is most apparent, and its applications are really fraught with the highest benefits to humanity, when exercised mainly by way of prevention. Its greatest achievements, and most honorable, are those by which evil has been averted from the frame, not those by which such evil is removed or palliated. My object now is to press upon the attention of my professional brethren the convictions which have been recorded here, and the grounds of which I have endeavored, briefly and imperfectly, to show both by reason and by practical illustration. If they are correct, the study of calculous diseases offers another example to those already existing of the extreme value, in relation to therapeutics, of all that tends to perfect the art of diagnosis—a principle the truth of which has been demonstrated by almost every improvement which has marked the progress of medicine and surgery during the present century.—(*London Lancet*, January 21, 1860.)

2. ANTIPHLOGISTIC POWERS OF MORPHIA, ILLUSTRATED BY ITS USE IN THE TREATMENT OF ACUTE INFLAMMATIONS OF THE SCLEROTIC AND IRIS. By *J. Zachariah Laurence, F.R.C.S., M.B., Lond.*, Surgeon to the South London Ophthalmic Hospital.—Perhaps the most (therapeutically) important distinction in the ophthalmiæ is, whether the conjunctiva or the sclerotic is the part principally affected. It would be foreign to my purpose to enter into the diagnosis of these two classes of inflammations. The most practical difference is in the nature of the pain. While in the conjunctival inflammation the pain is generally less severe, ("pricking or scalding,") more superficial, and referred by the patient to the eyelids; on the other hand, the sclerotic inflammation is characterized by a generally very severe, (occasionally agonizing,) often nocturnal pain, (rendering the patient quite sleepless,) of a deep-seated throbbing or dead character, and referred to the eyeball, eyebrow, temple, and head generally. Now the acknowledged and accepted treatment of the day of these cases of sclerotitis and iritis consists in a selection or combination of bleeding, leeching, cupping, blistering, and mercurialization.

I was first induced to try the effect of the morphia, (in Case 1,) rather with a view of relieving the excessive pain, than from the hope of any further result; but finding, to my great surprise, that not only was the pain relieved, but the disease itself on the decline, I continued the morphia then with a view of really testing its antiphlogistic powers; and, meeting with success, have administered the same remedy in other cases with good results. Some of these cases I now submit as the evidence of the antiphlogistic powers of morphia; and, the plan of treatment being different from that generally employed, have given them with a detail I should, under other circumstances, not have entered into.

*Case* 1.—*Acute Sclerotitis; Morphia Treatment; Decline of the Disease in about four-and-twenty hours.*—S. S., a middle-aged woman, was admitted to the South London Ophthalmic Hospital, on November 3, 1858. The sclerotic was intensely injected, the conjunctiva slightly; the "sclerotic zone" well marked. She suffered such severe shooting pain in the eyeball, eyebrow, and infra-orbital region, as to render her quite sleepless.

November 3.—℞ Morph. hydrochlor. gr. ¼, every third hour. Warm water fomentations to the eye.

6th.—Took the morphia regularly up to four P.M. yesterday, when she took the last powder. Toward the evening of the fourth the pain in the eye began to abate; now she feels but a slight aching in the eye on exposure to light. The sclerotic vascularity has considerably diminished. She now recovered rapidly under the treatment of a slight conjunctivitis.

*Case* 2.—*Acute Sclerotitis; Morphia Treatment; Decline of the Disease in less than twelve hours.*—H. B., an elderly, but strong man, admitted to the St. Marylebone Dispensary, July 27, 1859. Sclerotitis of a week's duration characterized by intense vascularity of the sclerotic, and a "sharp, burning" pain in the eyeball and forehead, with nocturnal exacerbation, rendering the patient sleepless. Suffering simultaneously from gout in the great toe. Has done nothing but foment the eye.

℞ Morph. hydrochlor. gr. ½, 3tia. quaque horâ. Warm water fomentations to the eye.

July 29.—Has taken the medicine as prescribed. Slept well, but not heavily, on the night of the twenty-seventh after midnight, when the hitherto severe pain in the eye left him. To-day, the vascularity of the tunics greatly diminished; the pain in the eyeball, brow, and forehead gone, leaving but a trifling pain at the side of the nose. Bowels have not acted since the twenty-seventh. To leave off the morphia and take an ounce of castor-oil.

August 1.—Perfectly recovered.

*Case* 3.—*Double Acute Iritis; Failure of Leeching and Mercurialization; Morphia Treatment; Decline of Disease within four-and-twenty hours.*—E. P. was admitted to the South London Ophthalmic Hospital, on August 27, 1859; during my absence from town, and up to September 10, when I first saw her, had been treated by leeching, blistering, mercurialization and belladonna lotion for the previous three weeks.

September 10.—Iris discolored; sclerotic deeply injected; pupils dilated (from the belladonna lotion;) humors muddy; complains of pains in the eyeballs and eyebrows, "like a rheumatic pain, of an overwhelming weight, of the light causing her great agony;" eyesight very dim.

℞ Morph. hydrochlor. gr. $\frac{1}{2}$, 4ta. quaque horâ. Warm water fomentations to the eyes.

14th.—Took the first dose of medicine on the night of the tenth. The pain abated, and, as she expressly stated, "very suddenly." She slept that night. On the following morning she could face the light much better. The medicine has made her feel very sick and drowsy. To-day she complains only of a little "pricking and shooting pain." Her eyes are still dim and weak, but the sclerotic injection is nearly gone.

28th.—Since the last report, has been taking the morphia in diminished doses, and subsequently a grain of quinine three times a day. Her eyes are to all appearances perfectly sound; nothing remains of her disease but a slight haziness of vision.

*Case* 4.—*Acute Sclerotitis; Morphia Treatment; Decline of the Disease in about seven hours.*—B. L., aged forty, a working engineer, was admitted to the South London Ophthalmic Hospital, on September 24, 1859. Five or six years ago he was struck on the now inflamed eye by something from a forge-fire. He recovered from the accident in about a month. The eye has been inflamed, as it is now, for the last three weeks. It presents all the usual signs of acute sclerotitis; great sclerotic vascularity, (the "sclerotic zone" well marked,) excessive lachrymation, great pain (especially at night, rendering him sleepless) referred to the eyeball, eyebrow, and temple, and compared by the patient to the sensation of a "weight hanging from his forehead, and pulling him down;" eyesight "foggy;" over inner part of the cornea a rust-colored opake speck, with a minute depression in its centre, evidenced the accident of five years back, but the most careful examination failed to detect any foreign body in the anterior chamber.

September 24.—℞ Morph. hydrochlor. gr. $\frac{1}{2}$, every third hour, watching its effects; warm water fomentations to the eye. Took the first dose about four P.M., felt sleepy about six P.M.; second dose about seven P.M.; the pain began then gradually to "die away;" the third dose about eleven P.M.; slept for three or four hours. The following day (Sunday) at noon but trifling pain was felt, and he slept soundly that night.

28th.—The case was reduced to one of slight conjunctivitis; all pain has left him; found his bowels confined from the medicine. To leave off the morphia, and take a purgative dose of calomel and colocynth, which completed the cure.

*Case* 5.—*Traumatic Acute Sclerotitis; Morphia Treatment; Decline of the Disease in less than four-and-twenty hours.*—C. H., aged forty-six, was on a Thursday evening engaged in Messrs. M.'s factory, pouring some molten iron into a sand mould, when a quantity of hot sand flew into his eye. He came to the South London Ophthalmic Hospital, on Saturday, October 1, 1859. With the exception of two minute particles of sand, which I removed with a spill of blotting-paper, all the sand had been removed by one of his fellow-workmen. I found him suffering from intense sclerotitis, marked by universal and high vascularity of the sclerotic and conjunctiva, great lachrymation and excessive pain in the eyeball, compared by the patient to the "prodding of a knife," and rendering him quite sleepless.

October 1.—℞ Morph. hydrochlor. gr. $\frac{1}{2}$, every third hour. Warm water fomentations.

Took the first dose of morphia about three P.M., and then regularly every three hours. It made him feel very drowsy, and that (Saturday) night he slept soundly. The violent pain was entirely gone on the following morning.

3d.—The case reduced to one of a simple conjunctivitis, and treated by a purgative dose of calomel, which completed the cure.

*Case* 6.—*Acute Sclerotitis; Failure of the Morphia Treatment; Recovery under Depletion and Mercurialization.*—E. S., aged forty-eight, applied at the South London Ophthalmic Hospital, on January 12, 1859. About twelve months before she lost the sight of the now inflamed eye by a cork from a soda-water bottle. The consequent inflammation of the eye lasted for only a few days; but three or four months afterwards her eyesight began gradually to fade, and she can now only distinguish the outlines (but not the colors) of objects with the injured eye. About three weeks before applying to the hospital she caught cold in the eye, which now presents the following signs: Intense sclerotic and conjunctival vascularity ("sclerotic zone" well marked); pupil central of medium size, angular, destitute of contractility. Pain intense, referred to the right eyeball and right side of the head, proceeding from the vertex downward to the level of the ala nasi.

January 12.—℞ Morph. hydrochl. gr. $\frac{1}{3}$, every fourth hour. Warm water fomentations to the eye.

15th.—Pain and other symptoms unabated. She recovered slowly under leeching, blistering, and mercurialization.

Whether in this case the deeply-diseased state of the eye, or the (too) small doses of the morphia, influenced the failure of the drug, must remain a matter of conjecture.

These cases I consider to establish an important practical fact, viz., that morphia is *per se* a powerful antiphlogistic,* capable of curing these acute inflammations of the eye, in which, up to the present time, blood-letting, blistering, and mercurialization have been considered necessary. As regards loss of blood, all will be agreed on the propriety of dispensing with it, where it can be done so with safety. Again, how constant an occurrence is it to see paroxysms of acute inflammations for a time apparently relieved by blood-letting, till the subsequent vascular reaction sets in, but to recur again and again, and require as many repetitions of this same objectionable remedy! I would further ask surgeons and physicians, What evidence have they that in the combination of mercury

---

* In all the cases mentioned, the patients had been using warm fomentations to the eyes before applying at the hospital.

and opium, given with a view of "putting the patient under the influence of mercury," as it is termed, it is not really the *opium* which does the good, and that the mercury and its action on the mouth may not be, to say the least, useless ?* And I would finally ask the physicians of this country to test the powers of morphia in the treatment of the acute inflammations of the internal organs of the body.

If we seek for an explanation of the above very remarkable action of morphia in reducing abnormal fullness of the vessels of the sclerotic, we may find it in the relations of pain to vascular congestion. Pain has generally been regarded rather as the effect than as the cause of the repletion of blood-vessels; but it is quite an open question whether or not in certain classes of cases the order of things may not be inverted. Such may be the case in the inflammations of the sclerotic we have just been discussing. That, on the other hand, vascular congestion may react as a cause of pain, is not improbable. The theory I would submit is that the action of morphia in these cases depends on its known power of reducing nervous irritability, which may be viewed as the primary cause of the inflammation. In these deep-seated inflammations of the eye this view is very much borne out by the seat of the pain; this will be found to follow strictly the branches of the fifth nerve; indeed, the precision with which the patients themselves localize the pain is very remarkable; while we have further evidence of the nervous nature of these cases in the intense watering of the eye, (dependent on irritation of the lachrymal branch of the fifth nerve.) In this way I conceive the irritation is propagated to the vessels through the intervention of the connections existing between the fifth and sympathetic nerves.—(*Medical Times and Gazette*, December 31, 1859.)

3. ON SYPHILITIC TUMORS OF THE TONGUE. By *M. Lagneau, Jr.* (Gazette Hebdomadaire, 1859, Nos. 32, 33, 35.)—In this paper M. Lagneau brings together the particulars of ten cases, some having been already recorded, and others having fallen under his own observation.

*Etiology.*—The affection has only been met with at an advanced stage of constitutional syphilis, some of the subjects being, nevertheless, otherwise in excellent health. Excepting one instance, all the cases occurred in males.

*Pathological Anatomy.*—The tumors are sometimes seated deep into the muscular substance of the organ, and at others very superficially; and their development commences most frequently at the base of the organ. The tumor may be single or exist in considerable numbers. Sometimes several isolated indurations in the process of growth become confounded together so as to constitute a single tumor. The size has varied from that of shot to that of a small walnut, and in a case occurring to M. Cloquet the tongue became so enlarged as to descend three inches below the chin. In general the form of the tumor is

* Again, mercury is presumed to have an "absorbing power" over plastic effusions, such as occur in acute iritis: here, too, it is a fair question whether the absorption of the inflammatory exudations is not rather a natural process, supervening on the cessation of the inflammation, (such as we daily see in the absorption of divided cataracts after the operation by solution, as soon as the inflammatory consequences of the operation have passed off,) than any, if I may be allowed the expression, "mercurial" process.

more or less rounded, and its color is gray or white, although this is not always the case. Of an almost cartilaginous hardness at first, as the tumor approaches the surface it presents more of a pasty or gummy consistence, and, on bursting, gives rise to ulceration. As these tumors do not prove fatal, their texture cannot well be judged of, but it is to be supposed that they do not materially differ in this respect from syphilitic tumors developed in the muscles and cellular tissue of other parts of the body. The excavated ulceration which follows the opening of the tumor is of variable depths, according to the position of the latter. It is of a more or less elongated form, with irregularly sharp cut edges, and presents a grayish bottom, covered with pseudomembranous exudation, and bleeding easily from contact of the teeth. Induration at first surrounds the base, but this gradually somewhat diminishes. When several of these ulcers become joined together a considerable portion of the substance of the tongue is destroyed, and a serious deformity remains even after cicatrization. When, however, the syphilitic tumors become arrested by suitable treatment while still in the condition of nodosities, they gradually lose their consistency, and leave behind them no signs of their former existence.

*Symptoms.*—Many of these have been already alluded to in detailing the appearances furnished by the tumors; of course, there is difficulty of speech and deglutition proportionate to the size of the tumors. The pain is usually but slight, or even does not exist, especially in the early stage. Although the occurrence is quite exceptional, the submaxillary glands sometimes undergo enlargement on the breaking out of ulceration. When there is great tumefaction and projection of the tongue, salivation is one of the consequences. The evolution of this affection of the tongue is eminently chronic, its commencement being often referred to a period of many months distant.

*Diagnosis.*—This is of the highest importance, for the affection has often been confounded with other lesions. Among these *cancer* is pre-eminent. Several patients, cured of frightful-looking syphilitic ulcers, have been regarded as instances of recovery from cancer of the organ, while the tongue has been amputated for reputed cancerous ulcerations which would have yielded to iodide of potassium. In distinguishing between the two affections we are greatly aided by the fact of the pre-existence or coexistence of other syphilitic symptoms, a fact which prevailed in nine out of ten cases here given. Cancer of the tongue is attended with lancinating pain, and its preferential seat is not the base but the point or edges of the tongue. It is usually single, while syphilitic tumors are mostly multiple. Cancer usually first shows itself as a hard, circumscribed, warty tumor, which is not the case with the syphilitic tumor, which assumes a more regularly roundish form. Consecutive glandular swellings are rare in the syphilitic affection; but cancer, when somewhat advanced, implicates the surrounding glandular tissue. The tendency of the syphilitic tumor is toward the surface, where it softens and ulcerates, while cancer involves the deep-seated as well as the superficial tissues. The ulceration from cancer is generally single and more or less fungous, while the ulcers following syphilitic tumors are generally multiple and excavated, with sharp, irregularly cut edges, and a grayish, diphtheritic, partially-gangrenous bottom. The early induration of their base gradually disappears as the plastic matter becomes softened and excreted at the surface of the ulcer. Cancerous engorgement is persistent, for, in proportion as the morbid product is destroyed by ulceration, the disease spreads to deeper and deeper parts. Both forms of ulcer bleed from contact with the teeth; but the syphilitic is less fungous and less vascular than the cancerous. (2) *Tubercle* going on to ulceration is not very uncommon in the tongue in phthisical or scrofulous subjects; and in such cases error may arise without much difficulty. These steatomatous tumors have usually less consistence than have the syphilitic. (3) *Callosities, hypertrophy of the epithelium, tylosis linguæ.* These terms indicate a condition described by MM. Ullmann and Buzenet as occurring on the tongue of inveterate smokers. The mucous membrane becomes adherent, and beneath it is deposited a plastic exudation, presenting a firm, whitish, insensible surface. This layer of plastic deposit and epithelial cells cracks and becomes detached in fragments, bringing into view a painful, irregular ulceration of grayish aspect. But, although this appearance somewhat resembles that caused by those syphilitic tumors which induce a whitish projection at the surface of the tongue, these latter are not found at the surface, but in the substance of the tongue. The ulcers which follow the detachment of these plastic deposits do not at all resemble the deep excavations which follow the syphilitic tumors. (4) *Hypertrophy* of the tongue; and (5) *Glossitis* are easily distinguished from syphilitic tumor. (6) *Primary chancre of the tongue* is usually easily distinguishable by the history of the case.

*Prognosis.*—This, in the case of suitable treatment, being had recourse to, is usually favorable; but, if this be too long delayed, the destructive ulceration which ensues will be followed by more or less permanent deformity.

*Treatment.*—Various forms of mercurial preparations have been used by different authors with success; and although the author believes that iodine is useful in some cases in which mercury has been already employed in vain, he does not agree with M. Ricord in the propriety of proscribing the latter, or even, as a general rule, in substituting iodide of potassium for it.—(*British and Foreign Medico-Chirurgical Review*, Jan. 1860.)

